# Gene Expression Signature of Fibroblast Serum Response Predicts Human Cancer Progression: Similarities between Tumors and Wounds

**DOI:** 10.1371/journal.pbio.0020007

**Published:** 2004-01-13

**Authors:** Howard Y Chang, Julie B Sneddon, Ash A Alizadeh, Ruchira Sood, Rob B West, Kelli Montgomery, Jen-Tsan Chi, Matt van de Rijn, David Botstein, Patrick O Brown

**Affiliations:** **1**Department of Dermatology, Stanford University School of MedicineStanford, CaliforniaUnited States of America; **2**Department of Biochemistry, Stanford University School of MedicineStanford, CaliforniaUnited States of America; **3**Department of Pathology, Stanford University School of MedicineStanford, CaliforniaUnited States of America; **4**Department of Genetics, Stanford University School of MedicineStanford, CaliforniaUnited States of America; **5**Howard Hughes Medical Institute, Stanford University School of MedicineStanford, CaliforniaUnited States of America

## Abstract

Cancer invasion and metastasis have been likened to wound healing gone awry. Despite parallels in cellular behavior between cancer progression and wound healing, the molecular relationships between these two processes and their prognostic implications are unclear. In this study, based on gene expression profiles of fibroblasts from ten anatomic sites, we identify a stereotyped gene expression program in response to serum exposure that appears to reflect the multifaceted role of fibroblasts in wound healing. The genes comprising this fibroblast common serum response are coordinately regulated in many human tumors, allowing us to identify tumors with gene expression signatures suggestive of active wounds. Genes induced in the fibroblast serum-response program are expressed in tumors by the tumor cells themselves, by tumor-associated fibroblasts, or both. The molecular features that define this wound-like phenotype are evident at an early clinical stage, persist during treatment, and predict increased risk of metastasis and death in breast, lung, and gastric carcinomas. Thus, the transcriptional signature of the response of fibroblasts to serum provides a possible link between cancer progression and wound healing, as well as a powerful predictor of the clinical course in several common carcinomas.

## Abstract 2

The transcriptional signature of the response of fibroblasts to serum provides a possible link between cancer progression and wound healing, as well as a predictor of the clinical course in several common cancers

## Introduction

Since the classic observations of the many histologic similarities between the tumor microenvironment and normal wound healing, it has been proposed that tumor stroma is “normal wound healing gone awry” (Dvorak 1986). During normal wound healing, coagulation of extravasated blood initiates a complex cascade of signals that recruit inflammatory cells, stimulate fibroblast and epithelial cell proliferation, direct cell migration, and induce angiogenesis to restore tissue integrity. Many of these normally reparative processes may be constitutively active in the tumor milieu and critical for tumor engraftment, local invasion, and metastasis to distant organs (Bissell and Radisky 2001). Indeed, keratinocytes from the wound edge transiently exhibit many similarities to their transformed counterparts in squamous cell carcinomas (Pedersen et al. 2003). Epidemiologically, chronic wound and inflammatory states are well-known risk factors for cancer development: the connection between cirrhosis and liver cancer, gastric ulcers and gastric carcinoma, and burn wounds and subsequent squa-mous cell carcinoma (so-called Majorlin's ulcer) are but a few examples. In the genetic blistering disorder recessive dystrophic epidermolysis bullosa, nearly 80% of the patients develop aggressive squamous cell carcinoma in their lifetime (Mallipeddi 2002), attesting to the powerful inductive environment of wounds for cancer development. In recent years, the roles of angiogenesis, extracellular matrix remodeling, and directed cell motility in cancer progression have been intensely studied (Bissell and Radisky 2001). Nonetheless, a comprehensive molecular view of wound healing and its relationship to human cancer is still lacking. Thus, there is currently no established method to quantify the risk of cancer from wounds diagnostically or to intervene therapeutically.

The complete sequence of the human genome and the advent of microarray technology have spurred a revolution in the classification and diagnosis of human cancers (Golub et al. 1999; Alizadeh et al. 2000; Perou et al. 2000; Sorlie et al. 2001; van 't Veer et al. 2002; Ramaswamy et al. 2003). By detailing the expression level of thousands of genes simultaneously in tumor cells and their surrounding stroma, gene expression profiles of tumors can provide “molecular portraits” of human cancers. The variations in gene expression patterns in human cancers are multidimensional and typically represent the contributions and interactions of numerous distinct cells and diverse physiological, regulatory, and genetic factors. Although gene expression patterns that correlate with different clinical outcomes can be identified from microarray data, the biological processes that the genes represent and thus the appropriate therapeutic interventions are generally not obvious. In this study, we explore an alternative strategy to infer physiologic mechanisms in human cancers. We began with a gene expression profile derived from a cell culture model of a physiological process. The in vitro expression profile is used to guide interpretation of publicly available gene expression data from human cancers and thereby test a specific hypothesis. In principle, this strategy allows one to connect the controlled and dynamic molecular perturbations possible in vitro with the complex biology of human clinical samples in a comprehensive and quantitative fashion.

Fibroblasts are ubiquitous mesenchymal cells in the stroma of all epithelial organs and play important roles in organ development, wound healing, inflammation, and fibrosis. Fibroblasts from each anatomic site of the body are differentiated in a site-specific fashion and thus may play a key role in establishing and maintaining positional identity in tissues and organs (Chang et al. 2002). Tumor-associated fibroblasts have previously been shown to promote the engraftment and metastasis of orthotopic tumor cells of many epithelial lineages (Elenbaas and Weinberg 2001). We previously observed that the genomic response of foreskin fibroblasts to serum, the soluble fraction of coagulated blood, represents a broadly coordinated and multifaceted wound-healing program that includes regulation of hemostasis, cell cycle progression, epithelial cell migration, inflammation, and angiogenesis (Iyer et al. 1999). We hypothesized that if one could identify a canonical gene expression signature of the fibroblast serum response, this signature might provide a molecular gauge for the presence and physiologic significance of the wound-healing process in human cancers.

## Results

### Identification of a Stereotyped Genomic Response of Fibroblasts to Serum

**Figure 1:**
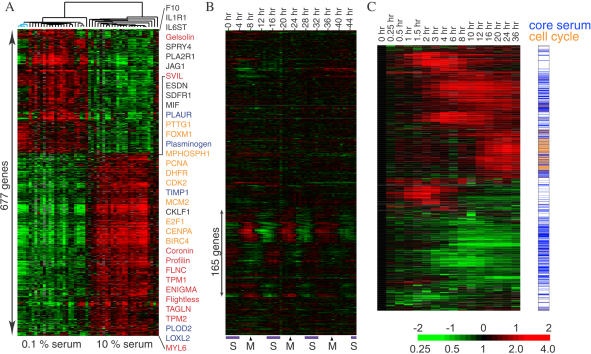
Identification and Annotation of a Common Serum Response in Fibroblasts (A) The fibroblast common serum response. Genes with expression changes that demonstrate coordinate induction or repression by serum in fibroblasts from ten anatomic sites are shown. Each row represents a gene; each column represents a sample. The level of expression of each gene in each sample, relative to the mean level of expression of that gene across all the samples, is represented using a red–green color scale as shown in the key; gray indicates missing data. Representative genes with probable function in cell cycle progression (orange), matrix remodeling (blue), cytoskeletal rearrangement (red), and cell–cell signaling (black) are highlighted by colored text on the right. Three fetal lung fibroblast samples, cultured in low serum, which showed the most divergent expression patterns among these samples (in part due to altered regulation of lipid biosynthetic genes [Chang et al. 2002]), are indicated by blue branches. (B) Identification of cell cycle-regulated genes in the common serum response signature. The expression pattern of each of the genes in (A) during HeLa cell cycle over 46 h after synchronization by double thymidine block is shown (Whitfield et al. 2002). Transit of cells through S and M phases during the timecourse, verified by flow cytometry, is indicated below. Approximately one-quarter of genes demonstrate a periodic expression patterns and are therefore operationally annotated as cell cycle genes; the remainder of the genes are used in further analyses to define the CSR. (C) Validation of annotation by temporal expression profiles. Timecourse of gene expression changes in a foreskin fibroblast culture after shifting from 0.1% to 10% FBS is shown. Global gene expression patterns were determined using cDNA microarrays containing 36,000 genes; genes whose transcript levels changed by at least 3-fold during the timecourse and those in (A) are displayed. The cell cycle genes identified in the analysis illustrated in (B) were found to have a distinct temporal expression pattern with coordinate upregulation at 12 h.

We previously observed that the global transcriptional response of fibroblasts to serum integrates many processes involved in wound healing (Iyer et al. 1999). Because fibroblasts from different anatomic sites are distinct differentiated cells with characteristic gene expression profiles (Chang et al. 2002), we investigated whether the genomic responses to serum varied significantly among fibroblasts cultured from different anatomic sites. Fifty fibroblast cultures derived from ten anatomic sites were cultured asynchronously in 10% fetal bovine serum (FBS) or in media containing only 0.1% FBS. Analysis of the global gene expression patterns, using human cDNA microarrays containing approximately 36,000 genes, revealed that although fibroblasts from different sites have distinctly different gene expression programs, they share a stereotyped gene expression program in response to serum (Figure 1A). Selection for genes that were concordantly induced or repressed by most types of fibroblasts yielded 677 genes, represented by 772 cDNA probes, of which 611 are uniquely identified by UniGene (http://www.ncbi.nlm.nih.gov/entrez/query.fcgi?db=unigene). This common genomic response to serum includes induction of genes that represent entry into and progression through the cell cycle (e.g., *E2F1*, *FOXM1*, *PTTG1*), induction of cell motility (e.g., *CORO1C*, *FLNC*), extracellular matrix remodeling (*LOXL2*, *PLOD2*, *PLAUR*), cell–cell signaling (*SDFR1*, *ESDN*, *MIF*), and acquisition of a myofibroblast phenotype (e.g., *TAGLN*, *TPM2*, *MYL6*). Analysis of the public Gene Ontology (GO) annotation of the fibroblast serum response genes confirmed a significant enrichment of genes involved in cell proliferation, blood coagulation, complement activation, secretory protein synthesis, angiogenesis, and proteolysis, reflecting the diverse roles that fibroblasts may play during wound healing (Worksheet 9 in Dataset S2).

One of the most consistent and important responses of human cells to serum is proliferation. Abnormal cell proliferation is also a consistent characteristic of cancer cells, irrespective of any possible involvement of a wound-healing response. We therefore sought to eliminate the contributions of genes directly related to cell proliferation, to improve the specificity of a genomic signature of the fibroblast serum response. To identify features directly related to cell cycle progression, we examined the expression pattern of these 677 genes during the cell cycle (in HeLa cells) (Whitfield et al. 2002). Despite the well-known role of serum as a mitogen, only one-quarter (165 out of 677 genes) of the fibroblast serum response genes showed periodic expression during the cell cycle (Figure 1B). The majority of the genes whose expression levels in fibroblasts showed the most consistent response to serum exposure do not appear simply to reflect cell growth or division; these 512 serum-responsive and cell cycle-independent genes are operationally defined as the fibroblast core serum response (CSR). Comparison of the common fibroblast serum response with a detailed analysis of the temporal program of gene expression following serum exposure in foreskin fibroblasts confirmed that the cell cycle genes and the CSR have distinct temporal profiles during serum stimulation and are thus distinguishable biological processes (Figure 1C).

### Expression of Fibroblast CSR in Human Cancers

**Figure 2:**
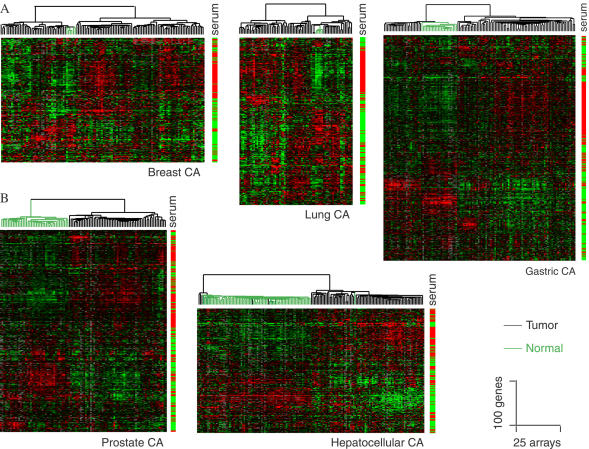
Survey of Fibroblast CSR Gene Expression in Human Cancers Expression patterns of available CSR genes in over 500 tumors and corresponding normal tissues were extracted, filtered as described in Materials and Methods, and organized by hierarchical clustering. The response of each gene in the fibroblast serum response is shown on the right bar (red shows activated; green shows repressed by serum). The strong clustering of the genes induced or repressed, respectively, in fibroblasts in response to serum exposure, based solely on their expression patterns in the tumor samples, highlights their coordinate regulation in tumors. The dendrograms at the top of each data display represent the similarities among the samples in their expression of the fibroblast CSR genes; tumors are indicated by black branches, normal tissue by green branches.

Because serum (as distinct from plasma and normal extracellular fluid) is encountered in vivo only at sites of tissue injury or remodeling and induces in fibroblasts a gene expression response suggestive of wound healing, we reasoned that expression of fibroblast CSR genes in tumors might gauge the extent to which the tumor microenvironment recapitulates normal wound healing. We examined the expression of genes comprising the fibroblast CSR in publicly available microarray data from a variety of human cancers and their corresponding normal tissues. To facilitate visualization and analysis, we organized the gene expression patterns and samples by hierarchical clustering (Eisen et al. 1998). Remarkably, we observed a predominantly biphasic pattern of expression for the fibroblast CSR in diverse cancers, including breast cancers, lung cancers, gastric cancers, prostate cancers, and hepatocellular carcinoma. Expression levels of genes that were activated by serum in fibroblasts varied coordinately in tumors, and genes that were repressed by serum in fibroblasts were mostly expressed in a reciprocal pattern (Figure 2).

In each of the tumor types examined, the expression pattern of the fibroblast CSR genes in normal tissues closely approximated that seen in quiescent fibroblasts cultured in the absence of serum (Figure 2). In prostate and hepatocellular carcinomas, all of the normal tissue samples had the serum-repressed signature and almost all of the tumors had the serum-induced signature, albeit with varying amplitude. In breast, lung, and gastric carcinomas, the common fibroblast serum response signature was clearly evident in some of the tumors and apparently absent in others, suggesting that a “wound-healing phenotype” was a variable feature of these cancers. We therefore classified breast, lung, and gastric cancer samples based on the pattern of expression of the genes that comprise the fibroblast CSR.

### Link between the Gene Expression Signature of Fibroblast Serum Response and Cancer Progression

**Figure 3:**
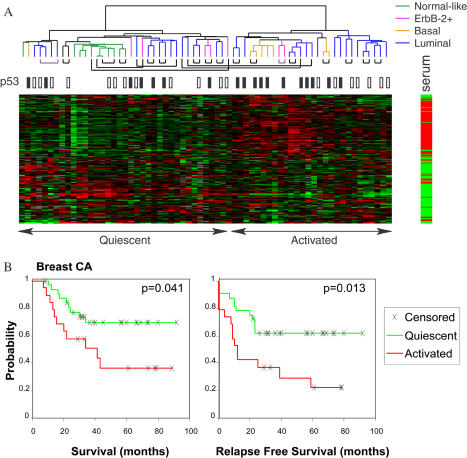
Context, Stability, and Prognostic Value of Fibroblast CSR in Breast Cancer (A) Expression patterns of CSR genes in a group of breast carcinomas and normal breast tissue previously described in Perou et al. (2000). Genes and samples were organized by hierarchical clustering. The serum response of each gene is indicated on the right bar (red shows induced; green shows repressed by serum). Note the biphasic pattern of expression that allows each tumor sample to be classified as “activated” or “quiescent” based on the expression of the CSR genes. The previously identified tumor phenotype (color code) and p53 status (solid black box shows mutated; white box shows wild-type) are shown. Pairs of tumor samples from the same patient, obtained before and after surgery and chemotherapy, are connected by black lines under the dendrogram. Two primary tumor–lymph node metastasis pairs from the same patient are connected by purple lines. (B) Kaplan–Meier survival curves for the two classes of tumors. Tumors with serum-activated CSR signature had worse disease-specific survival and relapse-free survival compared to tumors with quiescent CSR signature. Similar results were obtained whether performing classification using all breast tumors in this dataset or just the 58 tumors from the same clinical trial (Sorlie et al. 2001).

To investigate the stability and consistency of the serum response signature in individual tumors and to explore its clinical implications, we examined CSR gene expression in a group of locally advanced breast cancers with extensive clinical and molecular data (Perou et al. 2000; Geisler et al. 2001; Sorlie et al. 2001). As shown in Figure 3A, the expression profiles of the CSR genes were biphasic, allowing a natural separation of these tumors into two classes. Interestingly, in 18 out of 20 paired tumor samples obtained from the same patients before and after excisional biopsy and chemotherapy, the CSR expression phenotypes were consistent between the two samples. Thus, the wound-related expression program appears to be an intrinsic property of each tumor and not easily extinguished. In a set of 51 patients with clinically matched disease and equivalent treatment (Sorlie et al. 2001), primary tumors with the activated CSR signature were significantly more likely to progress to metastasis and death in a 5-y follow-up period (*p* = 0.013 and 0.041, respectively) (Figure 3B). Using an alternative analytic approach, classifying each sample by the Pearson correlation between tumor and fibroblast expression patterns of the fibroblast CSR genes, also reproduced the identification of two classes of samples with differing clinical outcomes (Worksheet 2 in Dataset S2). A gene expression pattern similar to the serum-activated program of fibroblasts is thus a powerful predictor of prognosis. Other significant prognostic factors in these same patients include tumor grade, estrogen receptor status, and tumor subtype based on gene expression profile (Geisler et al. 2001; Sorlie et al. 2001). Tumor stage, lymph-node status, and p53 status were not statistically significant predictors of survival in these patients (*p* = 0.13, 0.79, 0.05, respectively). A “basal-like” subtype of breast cancer, characterized by molecular similarities of the tumor cells to basal epithelial cells of the normal mammary duct and associated with a particularly unfavorable prognosis (Sorlie et al. 2001), was significantly associated with a gene expression pattern resembling the fibroblast CSR: six of seven basal-like breast cancers had the “serum-activated” gene expression signature (*p* = 0.0075, Fisher's exact test). Thus, the presence or absence of the wound-like phenotype may be linked to intrinsic features of the tumor cells.

We considered the possibility that the observed phenomenon may be simply a reflection of the number of fibroblasts in tumor samples. Perhaps tumors that are infiltrative or otherwise worrisome clinically would demand a wide margin of excision that would include more fibroblasts in the resultant samples. However, classification of breast cancers using the top 1% most highly expressed fibroblast genes (which include a number of extracellular matrix genes and have been previous observed as the “stroma signature” [Perou et al. 2000]) showed no relationship between the generic fibroblast signature and clinical outcome (*p* = 0.75; Worksheet 1 in Dataset S2). Thus, the prognostic value of the fibroblast CSR likely reflects the physiologic state of the tumor microenvironment and not just the number of fibroblasts in tumor stroma. Similarly, although the mitotic index is an established criterion of tumor grade, classification of these tumors based on expression of cell cycle genes (specifically, all S and G2/M phase genes identified by Whitfield et al. [2002]) only had moderate prognostic value (*p* = 0.08; Worksheet 1 in Dataset S2). This result also suggests that the prognostic value of the fibroblast CSR is unlikely to be accounted for by the incomplete annotation and removal of genes representing cell growth or division.

**Figure 4:**
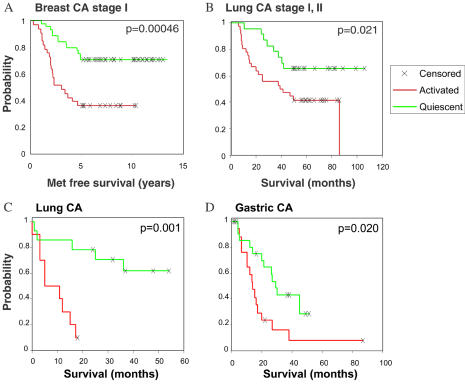
Prognostic Value of Fibroblast CSR in Epithelial Tumors Kaplan–Meier survival curves of tumors stratified into two classes using the fibroblast CSR are shown for stage I and IIA breast cancer (van 't Veer et al. 2002) (A), stage I and II lung adenocarcinoma (Bhattacharjee et al. 2001) (B), lung adenocarcinoma of all stages (Garber et al. 2001) (C), and stage III gastric carcinoma (Leung et al. 2002) (D).

To extend and validate these results, we tested the prognostic power of the fibroblast CSR signature in independent datasets and different kinds of human cancer (Figure 4). Using published DNA microarray data from a study of gene expression patterns in a group of 78 early (tumor smaller than 5 cm, stage I and IIA) breast cancer patients (van 't Veer et al. 2002), we could segregate the patients into two groups based on expression of the fibroblast CSR genes in the biopsy samples. Tumors with the serum-induced signature had a significantly increased risk of metastasis over 5 y (*p* = 0.00046) (Figure 4A). Multivariate Cox proportional hazard analysis confirmed that the CSR classification is a significant independent predictor (*p* = 0.009); the serum-induced gene expression signature was associated with a 3.3-fold relative risk of breast cancer metastasis within 5 y of diagnosis. In the two breast cancer datasets examined, approximately 50% of the CSR genes demonstrated significant differences in expression between the activated and quiescent groups of samples, but permutation and 10-fold balanced leave-one-out analyses revealed that the correct classification can be accomplished using as few as 6% of CSR genes (Worksheets 10–12 in Dataset S2). Thus, the expression pattern of the CSR genes provides a robust basis for predicting tumor behavior. Similarly, in analysis of published DNA microarray data from 62 patients with stage I and II lung adenocarcinomas (Bhattacharjee et al. 2001), tumors with the serum-induced signature were associated with significantly higher risk of death compared to tumors with the serum-repressed signature (*p* = 0.021) (Figure 4B). These results suggest that presence or absence of a wound-like phenotype in these cancers, with its prognostic implication for their metastatic potential, may be determined at an early stage in their development. In a second, independent group of lung adenocarcinomas of all stages (Garber et al. 2001), tumors with the fibroblast serum-induced signature were associated with a significantly worse prognosis (*p* = 0.0014) (Figure 4C). A significant correlation between advanced stage and the serum-induced signature was also apparent in this dataset. Finally, in 42 patients with stage III gastric carcinomas, all treated with gastrectomy alone (Leung et al. 2002), tumors with the activated CSR signature were again associated with shorter survival (*p* = 0.02) (Figure 4D). These results suggest that a wound-healing phenotype, reflected in the expression of a set of serum-inducible genes in fibroblasts, is strongly linked to progression of diverse human carcinomas and can provide valuable prognostic information even at an early stage in the natural history of a cancer.

For many other cancers, simple stratification based on expression of genes in the fibroblast CSR gene set is unlikely to be predictive of outcome. The dramatic differences in cellular composition and architecture among the tissues in which cancers can arise may influence the role that a wound-healing response can play in their progression. For example, lymphoma cells proliferate in the specialized microenvironment of lymph nodes and bone marrow, and the “stromal” cells in the central nervous system, predominantly astrocytes and microglia, are markedly different from those associated with most epithelial tissues. Indeed, in our initial analysis, the pattern of expression of the fibroblast CSR genes failed to stratify the outcomes in diffuse large B-cell lymphoma (Rosenwald et al. 2002), medulloblastoma (Pomeroy et al. 2002), and glioblastoma multiforme (M. Diehn and P. O. Brown, unpublished data).

### Histological Architecture of CSR Gene Expression in Tumors

**Figure 5:**
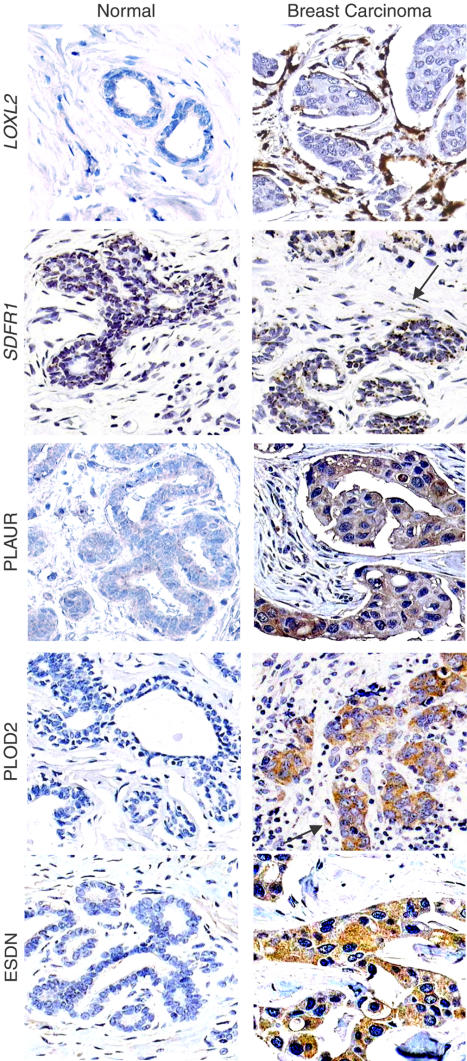
Histological Architecture of CSR Gene Expression in Breast Cancer Representative ISH of *LOXL2* and *SDFR1* and IHC of PLOD2, PLAUR, and ESDN are shown (magnification, 200×). Panels for *LOXL2*, PLAUR, PLOD2, and ESDN represent cores of normal and invasive ductal breast carcinoma from different patients on the same tissue microarray. Panels for *SDFR1* demonstrate staining in adjacent normal and carcinoma cells on the same tissue section. Arrows highlight spindle-shaped stromal cells that stain positive for *SDFR1* and PLOD2. No signal was detected for the sense probe for ISH or for control IHC without the primary antibody.

Both to validate the DNA microarray results and to investigate the histological architecture of CSR gene expression in tumors, we examined the expression patterns of five CSR genes implicated in extracellular matrix remodeling and cell–cell interaction, using tissue microarrays containing hundreds of breast carcinoma tissues. *PLAUR*, also known as urokinase-type plasminogen activator receptor, is a well-characterized receptor for matrix-degrading proteases that has been implicated in tumor cell invasion (Blasi and Carmeliet 2002; Sidenius and Blasi 2003). *LOXL2* is a member of a family of extracellular lysyl oxidases that modify and cross-link collagen and elastin fibers (Akiri et al. 2003). *PLOD2* is a member of the lysyl hydroxylase family that plays important roles in matrix cross-linking and fibrosis (Van Der Slot et al. 2003). *SDFR1*, previously named gp55 and gp65, encodes a cell surface protein of the immunglobulin superfamily that regulates cell adhesion and process outgrowth (Clarke and Moss 1994; Wilson et al. 1996). *ESDN* is a neuropilin-like cell surface receptor that was also previously found to be upregulated in metastatic lung cancers (Koshikawa et al. 2002). All five of these genes were included in the fibroblast CSR gene set by virtue of their induction by serum in fibroblasts (see Figure 1). Anti-PLAUR antibody is commercially available and served as a positive control. We prepared specific riboprobes for *LOXL2* and *SDFR1* and generated affinity-purified anti-peptide antibodies to PLOD2 and ESDN to detect the predicted protein products. As shown in Figure 5, PLAUR, *LOXL2,* PLOD2, and ESDN were not detectably expressed in normal breast tissue; *SDFR1* was expressed at a low level in normal breast epithelial cells (*n* = 11). In contrast, all five genes were induced in a significant fraction of invasive ductal carcinomas of the breast. As previously reported (Costantini et al. 1996), PLAUR protein is expressed in both tumor cells and peritumoral stroma (70 out of 96, 73% positive) (Figure 5). PLOD2 protein and *SDFR1* mRNA were detected in breast carcinoma cells and in a small but consistent fraction of peritumor stroma cells (78 out of 100, 78% positive, and 55 out of 79, 70% positive, respectively). ESDN protein was detected exclusively in breast carcinoma cells (69 out of 112, 62% positive). In contrast, *LOXL2* mRNA was abundant in peritumoral fibroblasts around invasive carcinomas (45 out of 106, 42% positive). LOXL2 protein has been previously reported to be expressed in normal mammary ducts and increased in invasive breast carcinoma cells (Akiri et al. 2003). Our data suggest that LOXL2 is primarily synthesized by peritumoral fibroblasts, but may act on or in the vicinity of epithelial cells during tissue remodeling. Collectively, these results suggest that the pathophysiology represented by expression of the fibroblast CSR genes in cancers represents a multicellular program in which the tumor cells themselves, tumor-associated fibroblasts, and perhaps diverse other cells in the tumor microenvironment are active participants.

## Discussion

The remarkable ability of a single physiological fluid—serum—to promote the growth and survival of diverse normal and cancer cells in culture suggests that there may be a conserved, programmed response to the molecular signals that serum provides. In vivo, serum as a physiological signal has a very specific meaning: cells encounter serum—the soluble fraction of coagulated blood—only in the context of a local injury. In virtually any tissue, a rapid, concerted multicellular response, with distinct physiological exigencies that evolve over minutes, hours, and days, is required to preserve the integrity of the tissue and often the survival of the organism. In response to a wound, many of the normal differentiated characteristics of the cells in the wounded tissue are temporarily set aside in favor of an emergency response. In wound repair, as in cancer, cells that ordinarily divide infrequently are induced to proliferate rapidly, extracellular matrix and connective tissues are invaded and remodeled, epithelial cells and stromal cells migrate, and new blood vessels are recruited. In all these respects, a wound response—and the characteristic physiological response to serum—would appear to provide a highly favorable milieu for cancer progression.

We defined a stereotyped genomic expression response of fibroblasts to serum, which reflects many features of the physiology of wound healing. When we examined the expression of these genes in human tumors, we found strong evidence that a wound-like phenotype was variably present in many common human cancers (including many that are not known to be preceded by chronic wounds) and was a remarkably powerful predictor of metastasis and death in several different carcinomas.

The proposed link between the fibroblast serum response signature and cancer progression raises many questions for additional studies. Perhaps most importantly, our results do not allow us to distinguish whether the wound-like phenotype has a functionally important role in tumor progression or merely serves as a marker for the underlying propensity of a cancer to progress and metastasize. However, at least three genes induced in the fibroblast serum response, *PLAUR*, *LOXL2*, and *MIF*, have been previously shown to increase cancer invasiveness or angiogenesis in animal xenograft models; each of these three genes has also been shown to play an important role in wound healing (Akiri et al. 2003; Nishihira et al. 2003; Sidenius and Blasi 2003). Thus, we are inclined to believe that coordinate induction of a wound-healing program in carcinomas contributes to tumor invasion and metastasis.

Several potential mechanisms might contribute to the wound-like gene expression pattern in cancers. In some cancers, ongoing local tissue injury, resulting from growth and dysfunctional behavior of the tumor cells, could continuously trigger a normal wound-healing response. The classic observation of deposited fibrin products in human tumors is consistent with this model (Dvorak 1986). Inflammatory cells, presumably recruited by tissue disorder, may amplify the wound response and contribute to tumor invasion in part by expression of metalloproteinases (Coussens et al. 2000; Daniel et al. 2003). The wound response might also be initiated directly by signals from the tumor cells (Fukumura et al. 1998), whose ability to activate an inappropriate wound-healing response—favorable to cell proliferation, invasion, and angiogenesis—might be strongly selected during cancer progression. The possibility that stromal cells might play a primary role in promoting a wound-like phenotype in some cancers is raised by studies showing that tumor-associated fibroblasts can enhance tumor engraftment and metastasis in animal models (Elenbaas and Weinberg 2001) and the demonstration in some cancers of genotypic abnormalities in tumor-associated fibroblasts (Kurose et al. 2002). Heterotopic interaction experiments, genetic models, and cell-culture models should enable these and other possible mechanisms to be investigated.

Our results illustrate the power of using gene expression data from specific cells or physiological and genetic manipulations to build an interpretive framework for the complex gene expression profiles of clinical samples (Lamb et al. 2003). Several prognostic models based on gene expression patterns have previously been identified from systematic DNA microarray profiles of gene expression in human cancers. Some of these prognostic gene expression profiles appear to reflect the developmental lineage of the cancer cells (Alizadeh et al. 2000; Sorlie et al. 2001; Pomeroy et al. 2002), some appear to reflect the activity of specific molecular determinants of tumor behavior (e.g., the activity of *PLA2G2A* in gastric cancer [Leung et al. 2002]), while still others represent the mechanistically agnostic results of machine-assisted learning (van 't Veer et al. 2002; Ramaswamy et al. 2003). Although they serve to identify many of the same tumors with unfavorable prognosis, the genes that define the fibroblast CSR overlap minimally with the genes previously used to predict outcome in the same cancers. For example, the fibroblast CSR involves only 20 out of 456 genes in an “intrinsic gene list” that can serve to segregate breast cancers into prognostically distinct groups (Perou et al. 2000) and four out of 128 genes that define the general metastasis signature reported by Ramaswamy et al. (2003). Only 11 genes are in common between the 231 gene van't Veer poor prognosis signature for breast cancer (van 't Veer et al. 2002) and the fibroblast CSR genes. The prognostic power of these different sets of genes illustrates the multidimensional variation in the gene expression programs in cancers and the complex interplay of many distinct genetic and physiological factors in determining the distinctive biology of each individual tumor. Our success in discovering a significant new determinant of cancer progression, using previously published and publicly available data, illustrates the richness of the data as a continuing source for future discoveries and the importance of unrestricted access to published research data (Roberts et al. 2001).

The signals and regulatory systems that normally initiate, sustain, and eventually shut down the physiological response to a wound remain to be identified and understood. Identification of the molecular control mechanisms in this pathway may pave the way to new cancer therapies or chemopreventative agents. For example, cyclooxygenase 2 is strongly induced in the response of fibroblasts to serum (Iyer et al. 1999), and platelet-derived growth factor is one of the principal molecular signals and mitogenic factors in serum. Platelet-derived growth factor receptor and cyclooxygenase 2 are inhibited by imatinib mesylate and nonsteroidal anti-inflammatory agents, respectively—two drugs with established efficacy in treating or preventing cancer (Bergers et al. 2003; Huls et al. 2003). Whether these or other small molecules might derive significant activity against cancer from their ability to inhibit a dysregulated wound-healing response will be an important question for future investigation.

## Materials and Methods

### 

#### Cells and tissue culture

Human primary fibroblasts from ten anatomic sites were cultured in 0.1% versus 10% FBS, as previously described (Chang et al. 2002). For the serum induction timecourse, foreskin fibroblasts CRL 2091 (American Type Culture Collection [ATCC], Manassas, Virginia, United States) were serum-starved for 48 h and harvested at the indicated timepoints after switching to media with 10% FBS, essentially as described in Iyer et al. (1999).

#### Microarray procedures

Construction of human cDNA microarrays containing approximately 43,000 elements, representing approximately 36,000 different genes, and array hybridizations were as previously described (Perou et al. 2000). mRNA was purified using FastTrack according to the manufacturer's instructions (Invitrogen, Carlsbad, California, United States). For the serum timecourse, RNA from all of the sampled timepoints were pooled as reference RNA to compare with RNA from individual timepoints as described in Iyer et al. (1999).

#### Data analysis

For defining a common serum response program in fibroblasts, global gene expression patterns in 50 fibroblast cultures derived from ten anatomic sites, cultured in the presence of 10% or 0.1% FBS, were characterized by DNA microarray hybridization (Chang et al. 2002). We selected for further analysis genes for which the corresponding array elements had fluorescent hybridization signals at least 1.5-fold greater than the local background fluorescence in the reference channel, and we further restricted our analyses to genes for which technically adequate data were obtained in at least 80% of experiments. These filtered genes were then analyzed by the multiclass Significance Analysis of Microarrays (SAM) algorithm (Tusher et al. 2001) to select a set of genes whose expression levels had a significant correlation with the presence of serum in the medium, with a false discovery rate (FDR) of less than 0.02%. The corresponding expression patterns were organized by hierarchical clustering (Eisen et al. 1998). Genes that were coordinately induced or repressed in response to serum in most samples (Pearson correlation, greater than 90%) were identified. This set of 677 genes, represented by 772 cDNA probes, of which 611 are uniquely identified by UniGene (http://www.ncbi.nlm.nih.gov/entrez/query.fcgi?db=unigene), was termed the common fibroblast serum response gene set. To identify the subset of these 677 genes whose variation in expression was directly related to cell cycle progression, we compared this set of genes to a published set of genes periodically expressed during the HeLa cell cycle (Whitfield et al. 2002). Because both datasets were generated using similar cDNA microarrays, we tracked genes by the IMAGE number of the cDNA clones on the microarrays. The majority of the genes in the fibroblast serum response gene set showed no evidence of periodic expression during the HeLa cell cycle. One hundred sixty-five genes, represented by 199 cDNA clones, overlapped with the cell cycle gene list; the remaining 512 genes, represented by 573 clones, of which 459 are uniquely identified in UniGene, was termed the CSR gene set.

The patterns of expression in human tumors of the 512 genes of the fibroblast CSR gene set were analyzed using data from published tumor expression profiles. Detailed methods and primary datasets are available as Datasets S1 and S2 and on our Web site (http://microarray-pubs.stanford.edu/wound). We used the Unigene unique identifier (build 158, release date January18, 2003) to match genes represented in different microarray platforms. For cDNA microarrays, genes with fluorescent hybridization signals at least 1.5-fold greater than the local background fluorescent signal in the reference channel (Cy3) were considered adequately measured and were selected for further analyses. For Affymetrix data, signal intensity values were first transformed into ratios, using for each gene the mean values of the normalized fluorescence signals across all the samples analyzed as the denominators (Bhattacharjee et al. 2001). The genes for which technically adequate measurements were obtained from at least 80% of the samples in a given dataset were centered by mean value within each dataset, and average linkage clustering was carried out using the Cluster software (Eisen et al. 1998). In each set of patient samples, the samples were segregated into two classes based on the first bifurcation in the hierarchical clustering dendrogram. For the datasets shown, the clustering and reciprocal expression of serum-induced and serum-repressed genes in the tumor expression data allowed two classes to be unambiguously assigned. Samples with generally high levels of expression of the serum-induced genes and low levels of expression of the serum-repressed genes were classified as “activated”; conversely, samples with generally high levels of expression of serum-repressed genes and low levels of expression of the serum-induced genes were classified as “quiescent.” Survival analysis by a Cox–Mantel test was performed in the program Winstat (R. Fitch Software).

#### In situ hybridization and immunohistochemistry

Digoxigenin-labeled sense and antisense riboprobes for *LOXL2* and *SDFR1* were synthesized using T7 polymerase-directed in vitro transcription (Iacobuzio-Donahue et al. 2002). Sense and antisense riboprobes for *SDFR1* were made from nucleotides 51–478 of IMAGE clone 586731 (ATCC #745139), corresponding to the last 388 nucleotides of the 3′ end of the coding sequence and 39 nucleotides of the 3′ untranslated region. Sense and antisense riboprobes for *LOXL2* were made from nucleotides 41–441 of IMAGE clone 882506 (ATCC #1139012), corresponding to the 3′ end of the coding sequence. In situ hybridization (ISH) results were considered to have appropriate specificity when we observed a strong, consistent pattern of hybridization of the antisense probe and little or no hybridization of the corresponding sense probe.

Immunohistochemical (IHC) staining was performed using Dako (Glostrup, Denmark) Envision Plus following the manufacturer's instructions. Anti-PLAUR antibody against whole purified human uPA–receptor protein (AB8903; Chemicon, Temecula, California, United States) was used at 1:200 dilution. Affinity-purified polyclonal antibody to PLOD2 was produced by immunizing rabbits with peptides EFDTVDLSAVDVHPN, coupled to keyhole limpet hemocyanin (KLH) (Applied Genomics, Inc., Sunnyvale, California, United States); affinity-purified antiserum was used for IHC at 1:25,000 dilution. Similarly, affinity-purified polyclonal antibody to ESDN was produced by immunizing rabbits with peptide DHTGQENSWKPKKARLKK coupled to KLH (Applied Genomics, Inc.) and used for IHC at 1:12,500 dilution. High-density tissue microarrays containing tumor samples were constructed as described in Kononen et al. (1998). ISH (Iacobuzio-Donahue et al. 2002) and IHC (Perou et al. 2000) were as reported. ISH and IHC images and data were archived as described in Liu et al. (2002).

## Supporting Information

Figure 1A can be interactively explored at http://microarray-pubs.stanford.edu/wound/. Raw datasets and all supporting data are also available at http://microarray-pubs.stanford.edu/wound/.

Dataset S1 Detailed Bioinformatic Methods Provides a description of microarray datasets, cross-platform mapping and data normalization, classification of cancers by fibroblast CSR genes and correlated clinical outcomes (Worksheets 1–8 in Dataset S2), the top 1% fibroblast genes in breast cancer prognosis (see Worksheet 1 in Dataset S2), cell cycle S and G2/M genes in breast cancer prognosis (see Worksheet 1 in Dataset S2), analysis of GO annotations of fibroblast serum response genes (see Worksheet 9 in Dataset S2), and the minimum number of CSR genes necessary for tumor classification (see Worksheets 10–12 in Dataset S2).
(120 KB DOC).

Dataset S2 Supporting Data Excel Worksheets of clinical and microarray data, as described in Dataset S1.
(736 KB XLS).

### Accession Numbers

The Locus Link (http://www.ncbi.nlm.nih.gov/LocusLink/) accession numbers for the genes discussed in this paper are *CORO1C* (Locus Link ID 23603), *E2F1* (Locus Link ID 1869), *ESDN* (Locus Link ID 131566), *FLNC* (Locus Link ID 2318), *FOXM1* (Locus Link ID 2305), *LOXL2* (Locus Link ID 4017), *MIF* (Locus Link ID 4282), *MYL6* (Locus Link ID 4637), *PLAUR* (Locus Link ID 5329), *PLOD2* (Locus Link ID 5352), *PTTG1* (Locus Link ID 9232), *SDFR1* (Locus Link ID 27020), *TAGLN* (Locus Link ID 6876), and *TPM2* (Locus Link ID 7169).

The accession numbers of the Gene Ontology (GO) (http://www.geneontology.org/) terms that appear in Dataset S1 are angiogensis (GO:0001525), blood coagulation (GO:0007596), complement activation (GO:0006956), immune response (GO:0006955), N-linked glycosylation (GO:0006487), protein translation (GO:0006445), and proteolysis and peptidolysis (GO:0006508).

## Abbreviations

CSR: core serum response
FBS: fetal bovine serum
FDR: false discovery rate
GO: Gene Ontology
IHC: immunohistochemistry
ISH: in situ hybridization
KLH: keyhole limpet hemocyanin
SAM: significance analysis of microarrays
