# Supplementary material for: Gene Expression Signature of Fibroblast Serum Response Predicts Human Cancer Progression: Similarities between Tumors and Wounds
Source: PLoS Biol. 2004 Jan 13;2(2):e7. doi: 10.1371/journal.pbio.0020007 (PMC314300; doi:10.1371/journal.pbio.0020007)
Supplement: Dataset S1 — Provides a description of microarray datasets, cross-platform mapping and data normalization, classification of cancers by fibroblast CSR genes and correlated clinical outcomes (Worksheets 1–8 in Dataset S2), the top 1% fibroblast genes in breast cancer prognosis (see Worksheet 1 in Dataset S2), cell cycle S and G2/M genes in breast cancer prognosis (see Worksheet 1 in Dataset S2), analysis of GO annotations of fibroblast serum response genes (see Worksheet 9 in Dataset S2), and the minimum number of CSR genes necessary for tumor classification (see Worksheets 10–12 in Dataset S2). (120 KB DOC). [file pbio.0020007.sd001.doc]

# **SUPPORTING INFORMATION**

For Chang *et al.* (2004) Cancer and a Wound Response: Gene Expression Signature of Fibroblast Serum Response Predicts Human Cancer Progression. **PLoS Biology**. DOI: DOI: 10.1371/journal/pbio.0020007

### I. Datasets

cDNA microarray data

A. **Molecular portrait of breast cancer**- 62 sporadic breast cancers and 3 pooled normal breast tissues, including 20 pairs of tumors obtained before and after excisional biopsy and doxorubicin-based chemotherapy and 2 pairs of primary tumor and lymph node metastasis. Published by (Perou et al., 2000).

1. **Locally advanced breast cancer**- 85 breast samples, consisting of 78 carcinomas, 3 fibroadenomas, and 4 normals. 40 of these tumor were previously profiled in Dataset A. A subset of 51 locally advanced primary breast cancers were all treated with excisional biopsy and doxorubicin-based chemotherapy. Clincal endpoint= relapse free survival and disease-specific survival. Published by (Sorlie et al., 2001).
2. **Lung cancer**- 67 sporadic primary lung carcinomas of different histologic types and stages, including 24 primary adenocarcinomas. 6 normal lung tissues were also profiled. Clinical endpoint= overall survival. Published by (Garber et al., 2001).
3. **Gastric cancer**-104 sporadic primary gastric carcinomas with > 5 year followup and 24 non-neoplastic gastric mucosa. All patients were treated with gastrectomy alone. Stage III presentation (n =42) was the most common and was analyzed for the clinical endpoint of overall survival. Published by (Leung et al., 2002).
4. **Diffuse large B cell lymphoma**- 240 DLCL patients with >5 year followup. Clinical endpoint= overall survival. Published by (Rosenwald et al., 2002).
5. **Hepatocellular carcinoma**- 156 HCC and non-cancerous liver tissues studied by (Chen et al., 2002).
6. **Prostate cancer**-100 prostate cancers and adjacent normal tissues profiled by Lapointe et al., manuscript submitted.

Rosetta ink jet oligonucleotide microarray data

1. **Early breast cancer**- 78 stage sporadic primary breast carcinomas < 5 cm diameter (stage I and IIA) with > 5 year clinical followup after lumpectomy. Clinical endpoint= metastasis. Data published by (van 't Veer et al., 2002).

Affymetrix Genechip data

1. **Early lung cancer**- 156 lung samples, including 127 sporadic primary adenocarcinomas of the lung, (62 of which were stage I and II), 12 suspected extrapulmonary metastases, and 17 normal lung samples with >4 year clinical followup. Clinical endpoint= overall survival. Data published by (Bhattacharjee et al., 2001)and stage I and II data selected by (Ramaswamy et al., 2003).
2. **Medulloblastoma**- 60 medulloblastomas with >5 year clinical followup. Clinical endpoint= overall survival. Published by (Pomeroy et al., 2002).

### II. Cross platform mapping and data normalization

**Breast Cancer Data (van’t Veer et al.)**: We downloaded and combined the raw microarray hybridization data for 78 Stage I breast tumors from the supplemental materials accompanying Van’t veer et al from <http://www.rii.com/publications/2002/vantveer.htm>). We then mapped each arrayed feature on the microarrays to the corresponding genes using BatchSOURCE (Diehn et al., 2003), where the 24,481 GenBank accessions provided by the authors were used as queries to retrieve UniGene identifiers (build #158, 1/15/2003). Since not all GenBank accessions are represented within UniGene, we could not map 636 (~2.6%) of the arrayed features in this manner. 456 of the 23845 Rosetta array elements that could be mapped corresponded to the fibroblast CSR genes present on our cDNA microarrays, and were used for subsequent analyses. Because the downloadable data were presented as 2-color ratios in log base 10 space, we simply transformed the measurements to log base 2 space to allow comparison to the spotted DNA microarray data. Consistent with the scheme employed for all 2-color hybridization arrays considered in this study, we filtered out genes with fewer than 80% data present (453 genes passed the filter). These data were then processed as detailed in section III below.

**Lung Adenocarcinoma (Bhattacharjee et al.):**

**Data access**: We downloaded raw microarray data (U95A series) for 156 specimens including 127 primary lung adenocarcinomas, 12 suspected extrapulmonary metastases from the lung, and 17 normal lung samples from the supplemental website accompanying Bhattacharjee et al (their ‘Dataset B’, available at <http://research.dfci.harvard.edu/meyersonlab/lungca/files/DatasetB_12600gene_Fig2order.txt>).

**Data processing:** Because the data provided by the authors were intensity measurements processed by a rank-invariant scaling scheme, we converted these intensities to normalized log-ratios to allow comparison of the corresponding measurements from cDNA microarrays. Specifically, following the protocol employed by Ramaswamy et al, we (1) considered all measurements regardless of Present (“P”) or Absent (“A”) call, (2) then applied a thresholding filter which arbitrarily sets values less than 20 to 20, and those above 16000 to 16000, and (3) then applied a variation filter such that we only considered those features which exhibited variation of at least 100 in intensity and which showed at least 3-fold difference in the intensity between the highest and lowest expression levels across the 156 microarrays (6349 of 12600 passed these criteria). Following these 3 steps, we then (1) generated ratios by mean centering the expression data for each gene (by dividing the intensity measurement for each gene on a given array by the average intensity of the gene across all 156 arrays), (2) then log-transformed (base 2) the resulting ratios, and (3) then median centered the expression data across arrays then across genes (2 iterations).

**UniGene mapping/CSR cross-referencing:** We next mapped the 12,454 probe sets (excluding control elements) represented on these U95A Affymetrix microarrays to the corresponding GenBank accessions of the mRNA targets, using the NetAffx resource (Liu et al., 2003) (<http://www.affymetrix.com/analysis/download_center.affx>) as well as “Table A” from the supplement to Ramaswamy et al ([Mets_Supplement_Information_110802_Final_SR.xls](http://www-genome.wi.mit.edu/mpr/publications/projects/Metastasis/Mets_Supplement_Information_110802_Final_SR.xls)). These accessions were then used in BatchSOURCE (Diehn et al., 2003) and LocusLink queries or to retrieve the corresponding UniGene cluster IDs (build #158); in this manner we mapped 11,963 (~96%) probe sets to 9,311 unique UniGene clusters. Of these mapped probe sets, 246 (corresponding to 212 unique UniGene clusters) had corresponding features represented in the CSR gene list, and were used for further analyses as described below.

**Medulloblastoma (Pomeroy et al.):**

**Data access**: we downloaded raw microarray data (HuGeneFL series) for 60 specimens from the supplemental website accompanying Ramaswamy et al. (their ‘Dataset E’, available at <http://www-genome.wi.mit.edu/mpr/publications/projects/Metastasis/DatasetE_medulloblastoma_outcome.res>).

**Data processing:** Because the data provided by the authors were intensity measurements processed by a linear scaling scheme (Ramaswamy et al., 2003), we converted these intensities to normalized log-ratios to allow comparison of the corresponding measurements from cDNA microarrays. Specifically, following the convention employed by Ramaswamy et al, we (1) considered all measurements regardless of Present (“P”) or Absent (“A”) call, and (2) then applied a thresholding filter which arbitrarily sets values less than 20 to 20, and those above 16,000 to 16,000. Following these steps, we then (1) generated ratios by mean centering the expression data for each gene (by dividing the intensity measurement for each gene on a given array by the average intensity of the gene across all 60 arrays), (2) then log-transformed (base 2) the resulting ratios, and (3) then median centered the expression data across arrays then across genes (2 iterations). Following these 2 steps, we then (1) generated ratios by mean centering the expression data for each gene (by dividing the intensity measurement for each gene on a given array by the average intensity of the gene across all 60 arrays), (2) then log-transformed (base 2) the resulting ratios, and (3) then median centered the expression data across arrays then across genes (2 iterations).

**UniGene mapping/CSR cross-referencing:** We next mapped the 7,129 probe sets represented on these HuGeneFL Affymetrix microarrays to the corresponding GenBank accessions of the mRNA targets, using the NetAffx resource (Liu et al., 2003) (<http://www.affymetrix.com/analysis/download_center.affx>) as well as “Table A” from the supplement to Ramaswamy et al ([Mets_Supplement_Information_110802_Final_SR.xls](http://www-genome.wi.mit.edu/mpr/publications/projects/Metastasis/Mets_Supplement_Information_110802_Final_SR.xls)). We retrieved surrogate accessions for probe sets designed from TIGR consensus sequences from Wong Lab website at Harvard University ([http://biosun1.harvard.edu/complab/dchip/common%20u95a_hu6800.xls](http://biosun1.harvard.edu/complab/dchip/common u95a_hu6800.xls)). These accessions were then used in BatchSOURCE (Diehn et al., 2003) and LocusLink queries to retrieve the corresponding UniGene cluster IDs (build #158); we supplemented these mappings with an annotation file from Jean-Marie Rouillard at the University of Michigan (<http://dot.ped.med.umich.edu:2000/ourimage/pub/shared/JMR_pub_affyannot.html> , file “Hu6800_annot.xls” downloaded 2/25/03) . We in this manner mapped 7,079 (~99%) probe sets to 5,691 unique UniGene clusters (Build #158). Of these mapped probe sets, 222 (corresponding to 181 unique UniGene clusters) had corresponding features represented in the CSR gene list, and were used for further analyses as described below.

III. Classification of Cancers by Fibroblast CSR genes and correlated clinical outcomes.

The patterns of expression in human tumors of the 512 genes of the fibroblast CSR gene set were analyzed using data from published tumor expression profiles listed above. We used IMAGE clone identifiers to follow the identity of cDNA probes of Stanford and NIH cDNA microarrays, and used Unigene unique identifier (build 158, release date Jan.18, 2003) to match genes represented in different microarray platforms. Transformation and normalization of expression data from different platforms are described above.

For cDNA microarray data, genes with fluorescent hybridization signals at least 1.5-fold greater than the local background fluorescent signal in the reference channel (Cy3) were considered adequately measured and were selected for further analyses. The genes for which technically adequate measurements were obtained from at least 80% of the samples in a given dataset were centered by mean value within each dataset, and average linkage clustering was carried out using the Cluster software (Eisen et al., 1998). In each set of patient samples, the samples were segregated into two classes based on the first bifurcation in the hierarchical clustering “dendrogram”. Unless otherwise noted, the clustering and reciprocal expression of serum-induced and serum repressed genes in the tumor expression data allowed two classes to be unambiguously assigned. Samples with generally high levels of expression of the serum-induced genes and low levels of expression of the serum-repressed genes, were classified as “activated”; conversely, samples with generally high levels of expression of serum-repressed genes and low levels of expression of the serum-induced genes were classified as “quiescent”. Survival analysis by Cox-Mantel test was performed in the program Winstat (R. Fitch Software).

For results shown in the paper, the expression data of CSR genes for each data set is provided in the cdt file and can be viewed using Treeview (http://rana.lbl.gov/EisenSoftware.htm). The correlated clinical data are available in Microsoft Excel worksheets as indicated below.

1. **Locally advanced breast cancer**. Results shown in Fig 3B. Dataset B.
2. **Classification of tumors using fibroblast CSR genes and correlated clinical outcomes** (Excel Worksheet 1). [A.Sorlie_breast_ca.cdt](http://microarray-pubs.stanford.edu/wound/Data).

The gene expression data of 58 samples (including 3 normal, 4 fibroadenomas, and 51 locally advanced breast cancers from the same clinical trial) were downloaded from Stanford Microarray Database ([http://smd.stanford.edu](http://smd.stanford.edu/)). Because the data were derived from several batches of microarrays (some containing different numbers of genes), the filtering criteria was relaxed to include genes with technically adequate data in 60% of experiments in order to preserve the expression data stemming from the larger arrays. 218 cDNA probes corresponding to CSR genes (henceforth genes) were present in this dataset and pass the filtering criteria. The expression pattern of these 218 genes were used for hierarchical clustering to define 2 classes were as described above. The 3 normal breasts and 4 fibroadenomas in this dataset were all identified as “quiescent”, along with 32 breast tumors. 19 tumors were classified as “activated.” The “activated” tumors demonstrated worse outcome in disease-specific survival and relapse free survival (p= 0.041 and 0.013, respectively). Applying CSR genes to the entire set of 85 breast carcinomas yielded similar classification result and prognostic stratification.

(ii) **Alternative strategy: Classification by Pearson correlation**. Excel worksheet 2.

To evaluate the validity of splitting tumor samples into two classes, we analyzed the expression pattern of CSR genes in the locally advanced breast cancers (Dataset B) by an alternative approach that quantifies the similarity of CSR gene expression in tumors vs. in cultured fibroblasts. The expression pattern of CSR genes in the 10 fibroblasts types cultured in 10% FBS was averaged to derive a single number for each gene. The Pearson correlation of the averaged fibroblast expression pattern with each of the breast cancer sample was then calculated. As shown in Excel worksheet 2, the Pearson correlation data demonstrated at least two groups of breast cancer samples: one group with expression patterns that have positive correlation to the fibroblast serum-induced expression pattern, and a second group with expression patterns that is anti-correlated with serum-induced expression. Plotting the Pearson correlations against uncensored survival time revealed that cancer samples with Pearson correlation greater than 0.2 had decreased survival and relapse-free survival. Using Pearson correlation of 0.2 as the cutoff, Cox-Mantel test confirmed that breast cancers with high correlation to fibroblast serum-induced expression of CSR genes indeed demonstrate poorer disease-specific survival and relapse free survival (p= 0.023 and 0.04, respectively).

1. **Lung cancer**- all stages. Result shown in Fig 4C. Dataset C. [B.Garber_lung_ca.cdt](http://microarray-pubs.stanford.edu/wound/Data). Excel worksheet 5

Gene expression data of 67 lung carcinomas and 6 normal lung tissues were downloaded from Stanford Microarray Database ([http://smd.stanford.edu](http://smd.stanford.edu/)). Genes with technically adequate measurement over 80% of experiments were selected; 338 cDNA probes corresponding to CSR genes (henceforth genes) were present in this dataset and pass the filtering criteria. The expression pattern of these 338 genes were used for hierarchical clustering to define 2 classes were as described above. The 6 normal lung tissues in this dataset were all identified as “quiescent”. Among 24 primary lung adenocarcinomas with adequate survival information, 10 tumors were classified as “activated” and 14 tumors were classified as “quiescent.” The “activated” tumors demonstrated worse overall survival (p= 0.001). There was an apparent association between the activated serum phenotype and advanced stage:7 out of 10 “activated” tumors had distant metastases at the time of presentation while only 3 of 14 patients with “quiescent” tumors had metastases at time of presentation.

1. **Gastric cancer**. Result shown in Fig. 4D. Dataset D. [C.Leung_gastric_ca.cdt](http://www.microarray-pubs.stanford.edu/wound/Data). Excel worksheet 6.

Gene expression data of 104 gastric carcinomas and 24 non-neoplastic gastric tissues were downloaded from Stanford Microarray Database ([http://smd.stanford.edu](http://smd.stanford.edu/)). Genes with technically adequate measurement over 80% of experiments were selected; 446 cDNA probes corresponding to CSR genes (henceforth genes) were present in this dataset and pass the filtering criteria. The expression pattern of these 446 genes were used for hierarchical clustering to define 2 classes were as described above. The 24 normal gastric tissues in this dataset were all identified as “quiescent”. Among 42 stage III primary gastric carcinomas with adequate survival information, 18 tumors were classified as “activated” and 24 tumors were classified as “quiescent.” The “activated” tumors demonstrated worse overall survival (p= 0.02).

1. **Diffuse large B cell lymphoma**. Dataset E. [D.Rosenwald_DLCL.cdt.](http://www.microarray-pubs.stanford.edu/wound/Data) Excel worksheet 7.

Gene expression data of 240 DLCL samples were downloaded from <http://llmpp.nih.gov/DLBCL/> . Genes with technically adequate measurement over 80% of experiments were selected; 198 cDNA probes corresponding to CSR genes (henceforth genes) were present in this dataset and pass the filtering criteria. The expression pattern of these 198 genes were used for hierarchical clustering to define 2 classes were as described above. We did not observe clear reciprocal expression of serum-induced and serum-repressed CSR genes within the samples. Thus, we took the first bifurcation of the hierarchical clustering dendrogram and classified samples as “A” or “B”, recognizing that the variation observed here may not have biological meaning. 110 samples were classified as “A” and 130 samples were classified as “B”. However, these two groups do not have significant difference in their overall survival (p=0.25).

1. **Hepatocellular carcinoma**. Result shown in Fig. 2. Dataset F. [E.Chen_HCC.cdt.](http://www.microarray-pubs.stanford.edu/wound/Data)

Gene expression data of 82 HCC and 74 non-neoplastic liver tissue were downloaded from Stanford Microarray Database ([http://smd.stanford.edu](http://smd.stanford.edu/)). Genes with technically adequate measurement over 80% of experiments were selected; 249 cDNA probes corresponding to CSR genes (henceforth genes) were present in this dataset and pass the filtering criteria. The expression pattern of these 249 genes were used for hierarchical clustering to define 2 classes were as described above. 73 out of 74 non-neoplastic liver tissues in this dataset were identified as “quiescent”. 77 out of 82 HCC samples were classified as “activated.” Because most tumors had the activated CSR phenotype, we did not analyze possible survival differences.

1. **Prostate cancer**. Result shown in Fig. 2. Dataset G. [F.Lapointe_Prostate_CA.cdt.](http://www.microarray-pubs.stanford.edu/wound/Data)

Gene expression data of 59 prostate cancers and 41 non-neoplastic prostate tissue were downloaded from Stanford Microarray Database ([http://smd.stanford.edu](http://smd.stanford.edu/)). Genes with technically adequate measurement over 80% of experiments were selected; 431 cDNA probes corresponding to CSR genes (henceforth genes) were present in this dataset and pass the filtering criteria. The expression pattern of these 431 genes were used for hierarchical clustering to define 2 classes were as described above. 40 out of 41 non-neoplastic prostate tissues in this dataset were identified as “quiescent”. 58 out of 59 HCC samples were classified as “activated.” Because most tumors had the activated CSR phenotype, we did not analyze possible survival differences.

1. **Early breast cancer**. Result shown in Fig. 4A. Dataset H. [G.vant_Veer_early_breast_CA.cdt.](http://www.microarray-pubs.stanford.edu/wound/Data) Excel worksheet 3.

Gene expression data of 78 stage I and IIA breast cancers were downloaded and processes as described above in section II. Genes with technically adequate measurement over 80% of experiments were selected; 453 CSR genes were present in this dataset and pass the filtering criteria. The expression pattern of these 453 genes were used for hierarchical clustering to define 2 classes were as described above. 33 tumors were classified as “activated” and 45 tumors were classified as “quiescent.” The “activated” tumors demonstrated worse metastasis-free survival over 10 years of followup (p= 0.00046).

1. **Early lung cancer**-stage I and II. Result shown in Fig. 4B. Dataset I. [H.Bhatacharjee_lung_CA.cdt.](http://www.microarray-pubs.stanford.edu/wound/Data) Excel worksheet 4

Gene expression data of 156 lung samples, including 62 stage I and II primary lung adenocarcinomas and 17 normal lung samples were downloaded and processes as described above in section II. Genes with technically adequate measurement over 80% of experiments were selected; 246 CSR genes were present in this dataset and pass the filtering criteria. The expression pattern of these 246 genes were used for hierarchical clustering to define 2 classes were as described above. 16 of 17 normal lung samples were classified as “quiescent.” Among the 62 stage I and II primary lung adenocarcinomas, 36 tumors were classified as “activated” and 26 tumors were classified as “quiescent.” The “activated” tumors demonstrated worse overall survival (p= 0.021).

1. **Medulloblastoma**. Dataset J. [I.Pomeroy_medullo.cdt](http://microarray-pubs.stanford.edu/wound/Data). Excel worksheet 8.

Gene expression data of 60 medulloblastoma samples were downloaded, transformed, and processed as described in section II. Genes with technically adequate measurement over 80% of experiments were selected; 222 CSR genes present in this dataset pass the filtering criteria. The expression pattern of these 222 genes were used for hierarchical clustering to define 2 classes were as described above. We did not observe clear reciprocal expression of serum-induced and serum-repressed CSR genes within the samples. Thus, we took the first bifurcation of the hierarchical clustering dendrogram and classified samples as “A” or “B”, recognizing that the variation observed here may not have biological meaning. 21 samples were classified as “A” and 39 samples were classified as “B”. However, these two groups do not have significant difference in their overall survival (p=0.65).

**IV.** **Top 1% Fibroblast genes in breast cancer prognosis**. Excel worksheet 1.

To identify genes that are constitutively and highly expressed in fibroblasts, the global gene expression data of 50 fibroblast cultures (Chang et al., 2002) was selected as follows. The median Cy5 fluorescence signal over background (representing expression of genes in fibroblasts) for each array element was filtered for regression > 0.6 over the element, Cy3 channel (representing reference RNA) signal > 1.5 fold over background, 80% informative data and variance less than 2 fold in 5 arrays over the 50 experiments. These filtering criteria identified 12959 array elements out of 44600 on the microarray. The Cy5 fluorescence signal of each gene was then averaged for the 50 experiments and ranked from high to low. Genes already identified as the universal fibroblast serum response were removed from this list. The top 1% this ranked gene list (122 out of 12213) was termed “top 1% fibroblast genes.”

To determine whether the top 1% fibroblast genes also had prognostic power in breast cancer, IMAGE clone number was used to map the genes in this list to array elements in breast cancer gene expression (Dataset B). 98 out of 122 genes were mapped. The extracted expression data was centered by mean, filtered for genes that were present for 80% of experiments, and the breast cancer samples were organized by the expression pattern of these genes as described above using hierarchical clustering. The top 1% fibroblast genes were up regulated in benign fibroadenomas, which is consistent with the known biology of fibroadenomas and confirms the selection of fibroblast-enriched genes. However, separation of 51 breast cancer samples into 2 groups based on this gene list did not identify a statistically significant survival difference between these two groups (p=0.75).

**V.** **Cell cycle S and G2/M genes in breast cancer prognosis**. Excel worksheet 1.

To compare the prognostic value of fibroblast CSR to a measure of cell proliferation, we chose to classify breast cancers Dataset B based on the expression pattern of all genes designated by Whitfield et al. as S or G2/M phase-specific (Whitfield et al., 2002). 535 out of 726 cDNA clones were mapped in the breast cancer data, and 224 out of 535 clones passed the filter criteria as above. The expression patterns and samples were organized by hierarchical clustering; the tumors overexpressing the S and G2/M phase signature demonstrated poorer outcome but with borderline stastistical significance in relapse free survival and overall survival (p= 0.06 and 0.08, respectively). Thus, although mitotic rate is one of the established criteria for tumor grade, the aggregate gene expression measurement of cell proliferation is not sufficiently robust to predict outcome. This result also indicates that the prognostic power of the fibroblast core serum response genes cannot be solely accounted for by the incomplete removal of genes representing cell cycle progression.

### VI. Analysis of Gene Ontology (GO) annotations of fibroblast serum response genes. Excel worksheet 9.

To confirm the interpretation that the common serum response of fibroblasts reflect their diverse roles in wound healing, we asked whether the serum response genes were enriched for biologic processes related to wound healing in the public Gene Ontology annotation database (<http://www.geneontology.org/>). The common fibroblast serum response were queried against the GO database using the program SOURCE (Diehn et al., 2003), and enrichment of GO-annotated biologic processes greater than expected by chance was calculated using a hypergeometric distribution model as previously described (Boldrick et al., 2002). Specifically, we compared the number of genes with a particular GO annotation in the query set (“sample succ”/sample num”) versus that ratio calculated for all genes on the microarray (“pop succ”/”pop num”)[Excel worksheet 9]. For genes in the unfiltered, common fibroblast serum response, the predominant biologic process annotations were related to cell proliferation. Once genes that have periodic expression during the cell cycle were removed (Fig. 1B,C), the enriched biologic processes include: blood coagulation (GO:0007596), angiogensis (GO:0001525), complement activation (GO:0006956), immune response (GO:0006955), proteolysis and peptidolysis (GO:0006508), and secretory protein synthesis such as N-linked glycosylation (GO:0006487) and protein translation (GO:0006445) [Excel worksheet 9]. This result reinforces the idea that the common transcriptional response of fibroblasts to serum *in vitro* recapitulates their multifaceted roles in wound healing *in vivo*.

**VII. Minimum number of CSR genes necessary for tumor classification.** Excel worksheets 10-12.

To understand how many of the CSR genes were driving the classification of tumors into two classes (Activated vs. Quiescent), we performed SAM analysis on the CSR gene expression patterns in two breast cancer datasets examined in this study (datasets B and H above). SAM is a permutation-based algorithm that calculates a false discovery rate (FDR) analogous to traditional p-values but has added advantages (Tusher et al., 2001). Of 217 CSR genes in the Sorlie dataset, 108 (50%) of the CSR genes were significantly different (FDR< 0.05) between the activated vs. the quiescent samples. Of the 456 CSR genes in the van’t Veer dataset, 237 genes (52%) were significantly different (FDR <0.05) between the activated and quiescent samples. Thus, a significant subset of the CSR genes are providing discriminating power to the tumor classification, highlighting the link between wound healing and cancer progression.

To address the level of redundancy of CSR genes in achieving tumor classification, we applied a shrunken centroid analysis in the program Prediction Analysis of Microarrays (PAM)(Tibshirani et al., 2002). Using a 10-fold balanced leave-one-out training and testing procedure, we discovered that as few as 35 CSR genes could recapitulate the classification in the Sorlie dataset, and as few as 38 CSR genes could recapitulate the classification in the van’t Veer dataset. In other words, a minimum of 6% of CSR genes may accomplish the diagnostic task. Because different published cancer gene expression datasets contain varying number of CSR genes, the robustness of the CSR gene classification underlies our success in using this one set of genes in stratifying prognosis in multiple types of human cancers. Nevertheless, we have noted that different subsets of CSR genes are more distinct in different types of cancers. Therefore, whether a small number of CSR genes can emerge as universal prognostic markers in epithelial cancers is an important issue for future studies.

### REFERENCES

Bhattacharjee, A., Richards, W. G., Staunton, J., Li, C., Monti, S., Vasa, P., Ladd, C., Beheshti, J., Bueno, R., Gillette, M.*, et al.* (2001). Classification of human lung carcinomas by mRNA expression profiling reveals distinct adenocarcinoma subclasses. Proc Natl Acad Sci U S A *98*, 13790-13795.

Boldrick, J. C., Alizadeh, A. A., Diehn, M., Dudoit, S., Liu, C. L., Belcher, C. E., Botstein, D., Staudt, L. M., Brown, P. O., and Relman, D. A. (2002). Stereotyped and specific gene expression programs in human innate immune responses to bacteria. Proc Natl Acad Sci U S A *99*, 972-977.

Chang, H. Y., Chi, J.-T., Dudoit, S., Bondre, C., van de Rijn, M., Botstein, D., and Brown, P. O. (2002). Diversity, topographic differentiation, and positional memory in human fibroblasts. Proc Natl Acad Sci U S A *99*, 12877-12882.

Chen, X., Cheung, S. T., So, S., Fan, S. T., Barry, C., Higgins, J., Lai, K. M., Ji, J., Dudoit, S., Ng, I. O.*, et al.* (2002). Gene expression patterns in human liver cancers. Mol Biol Cell *13*, 1929-1939.

Diehn, M., Sherlock, G., Binkley, G., Jin, H., Matese, J. C., Hernandez-Boussard, T., Rees, C. A., Cherry, J. M., Botstein, D., Brown, P. O., and Alizadeh, A. A. (2003). SOURCE: a unified genomic resource of functional annotations, ontologies, and gene expression data. Nucleic Acids Res *31*, 219-223.

Eisen, M. B., Spellman, P. T., Brown, P. O., and Botstein, D. (1998). Cluster analysis and display of genome-wide expression patterns. Proc Natl Acad Sci U S A *95*, 14863-14868.

Garber, M. E., Troyanskaya, O. G., Schluens, K., Petersen, S., Thaesler, Z., Pacyna-Gengelbach, M., van de Rijn, M., Rosen, G. D., Perou, C. M., Whyte, R. I.*, et al.* (2001). Diversity of gene expression in adenocarcinoma of the lung. Proc Natl Acad Sci U S A *98*, 13784-13789.

Leung, S. Y., Chen, X., Chu, K. M., Yuen, S. T., Mathy, J., Ji, J., Chan, A. S., Li, R., Law, S., Troyanskaya, O. G.*, et al.* (2002). Phospholipase A2 group IIA expression in gastric adenocarcinoma is associated with prolonged survival and less frequent metastasis. Proc Natl Acad Sci U S A *99*, 16203-16208.

Liu, G., Loraine, A. E., Shigeta, R., Cline, M., Cheng, J., Valmeekam, V., Sun, S., Kulp, D., and Siani-Rose, M. A. (2003). NetAffx: Affymetrix probesets and annotations. Nucleic Acids Res *31*, 82-86.

Perou, C. M., Sorlie, T., Eisen, M. B., van de Rijn, M., Jeffrey, S. S., Rees, C. A., Pollack, J. R., Ross, D. T., Johnsen, H., Akslen, L. A.*, et al.* (2000). Molecular portraits of human breast tumours. Nature *406*, 747-752.

Pomeroy, S. L., Tamayo, P., Gaasenbeek, M., Sturla, L. M., Angelo, M., McLaughlin, M. E., Kim, J. Y., Goumnerova, L. C., Black, P. M., Lau, C.*, et al.* (2002). Prediction of central nervous system embryonal tumour outcome based on gene expression. Nature *415*, 436-442.

Ramaswamy, S., Ross, K. N., Lander, E. S., and Golub, T. R. (2003). A molecular signature of metastasis in primary solid tumors. Nat Genet *33*, 49-54.

Rosenwald, A., Wright, G., Chan, W. C., Connors, J. M., Campo, E., Fisher, R. I., Gascoyne, R. D., Muller-Hermelink, H. K., Smeland, E. B., Giltnane, J. M.*, et al.* (2002). The use of molecular profiling to predict survival after chemotherapy for diffuse large-B-cell lymphoma. N Engl J Med *346*, 1937-1947.

Sorlie, T., Perou, C. M., Tibshirani, R., Aas, T., Geisler, S., Johnsen, H., Hastie, T., Eisen, M. B., van de Rijn, M., Jeffrey, S. S.*, et al.* (2001). Gene expression patterns of breast carcinomas distinguish tumor subclasses with clinical implications. Proc Natl Acad Sci U S A *98*, 10869-10874.

Tibshirani, R., Hastie, T., Narasimhan, B., and Chu, G. (2002). Diagnosis of multiple cancer types by shrunken centroids of gene expression. Proc Natl Acad Sci U S A *99*, 6567-6572.

Tusher, V. G., Tibshirani, R., and Chu, G. (2001). Significance analysis of microarrays applied to the ionizing radiation response. Proc Natl Acad Sci U S A *98*, 5116-5121.

van 't Veer, L. J., Dai, H., van de Vijver, M. J., He, Y. D., Hart, A. A., Mao, M., Peterse, H. L., van der Kooy, K., Marton, M. J., Witteveen, A. T.*, et al.* (2002). Gene expression profiling predicts clinical outcome of breast cancer. Nature *415*, 530-536.

Whitfield, M. L., Sherlock, G., Saldanha, A. J., Murray, J. I., Ball, C. A., Alexander, K. E., Matese, J. C., Perou, C. M., Hurt, M. M., Brown, P. O., and Botstein, D. (2002). Identification of genes periodically expressed in the human cell cycle and their expression in tumors. Mol Biol Cell *13*, 1977-2000.
